# Cut-off Value of Random Blood Glucose among Asian Indians for Preliminary Screening of Persons with Prediabetes and Undetected Type 2 Diabetes Defined by the Glycosylated Haemoglobin Criteria

**DOI:** 10.33696/diabetes.1.009

**Published:** 2019

**Authors:** Priscilla Susairaj, Chamukuttan Snehalatha, Arun Raghavan, Arun Nanditha, Ramachandran Vinitha, Krishnamoorthy Satheesh, Desmond G Johnston, Nicholas J Wareham, Ambady Ramachandran

**Affiliations:** 1 India Diabetes Research Foundation and Dr. A. Ramachandran’s Diabetes Hospitals, Chennai, India; 2 Faculty of Medicine, Imperial College, London, UK; 3 Medical Research Council Epidemiology Unit, Institute of Metabolic Science, University of Cambridge, UK

**Keywords:** Random blood glucose, Screening for diabetes, Prediabetes, Glycosylated hemoglobin

## Abstract

**Aim:**

The increased morbidity and mortality due to type 2 diabetes can be partly due to its delayed diagnosis. In developing countries, the cost and unavailability of conventional screening methods can be a setback. Use of random blood glucose (RBG) may be beneficial in testing large numbers at a low cost and in a short time in identifying persons at risk of developing diabetes. In this analysis, we aim to derive the values of RBG corresponding to the cut-off values of glycosylated hemoglobin (HbA1c) used to define prediabetes and diabetes.

**Methods:**

Based on their risk profile of developing diabetes, a total of 2835 individuals were screened for a large diabetes prevention study. They were subjected to HbA1c testing to diagnose prediabetes and diabetes. Random capillary blood glucose was also performed. Correlation of RBG with HbA1c was computed using multiple linear regression equation. The optimal cut-off value for RBG corresponding to HbA1c value of 5.7% (39 mmol/mol), and ≥ 6.5% (48 mmol/mol) were computed using the receiver operating curve (ROC). Diagnostic accuracy was assessed from the area under the curve (AUC) and by using the Youden’s index.

**Results:**

RBG showed significant correlation with HbA1c (r=0.40, p<0.0001). Using the ROC analysis, a RBG cut-off value of 140.5 mg/dl (7.8 mmol/L) corresponding to an HbA1c value of 6.5% (48mmol/mol) was derived. A cut-off value could not be derived for HbA1c of 5.7% (39 mmol/mol) since the specificity and sensitivity for identifying prediabetes were low.

**Conclusion:**

Use of a capillary RBG value was found to be a simple procedure. The derived RBG cut-off value will aid in identifying people with undiagnosed diabetes. This preliminary screening will reduce the number to undergo more cumbersome and invasive diagnostic testing.

## Introduction

Type 2 diabetes (T2D) remains undiagnosed for many years in large number of persons living in developing countries [1]. The cost of diagnostic tests and the invasive procedures involved in conventional screening methods remain a major setback to timely testing. According to the recent estimates by the International Diabetes Federation (IDF), there are more than 42.2 million people with undiagnosed diabetes only in India [2], which accounts for more than half the number living with diabetes. Recommendations have been given by the World Health Organization (WHO) [3] and American Diabetes Association (ADA) [4] on screening strategies for high risk groups. Previously reported studies in the Western [5,6] and Chinese Populations [7] have evaluated cut-off points using fasting plasma glucose (FPG), HbA1c and random blood glucose (RBG) to define both prediabetes and undiagnosed diabetes. A large community-based screening program in India has studied the correlation of random capillary blood glucose with oral glucose tolerance test (OGTT) values to define cut-points for identifying diabetes and prediabetes [8]. Screening using random capillary blood glucose offers great benefits for testing large numbers, at low cost and in a short time. However, definitive cut-off points for RBG corresponding to the respective glycated hemoglobin (HbA1c) values for prediabetes and diabetes specific for Asian Indians are not clearly defined. In this analysis, we aim to derive these cut-off values of RBG corresponding to HbA1c used to define prediabetes (5.7%, 39 mmol/mol) and diabetes (6.5%, 48 mmol/mol) [9].

## Materials and Methods

This is a post – hoc analysis of the primary prevention trial done in South India (Clinical Trials.Gov No. NCT01570946, Clinical Trials Registry – India Number: CTRI/2014/07/004799). The study design, recruitment strategy and the outcome of screening were described previously [10,11]. Briefly, the study cohort; men and women aged 35 to 55 years were prescreened for risk factors of diabetes using non-invasive diabetes risk assessment and then screened using HbA1c testing. Persons at a higher risk of conversion to diabetes, having HbA1c values between 6.0% (42 mmol/mol) and <6.5% (48 mmol/mol) were enrolled in the primary prevention study. They were randomized to receive either standard care with lifestyle advice at baseline or to the intervention arm to receive frequent text messages on their mobile phones on healthy lifestyle practices, in addition to the above. The study was approved by the Ethics Committee of India Diabetes Research Foundation on 28^th^ February 2012.

Recruitment of study participants was conducted between April 2012 and September 2013. From the 15 study sites a total of 6030 persons were identified to undergo initial prescreening using a non-invasive questionnaire method. At the prescreening, 2835 individuals were identified as having ≥ 3 risk factors for diabetes and were selected for the next stage of screening using HbA1c testing. The HbA1c test was performed using a point-of-care device (Bio-Rad in2it™ (I) System). This instrument has been standardized to the recommendations of the Diabetes Control and Complications Trial and certified by the National Glycohemoglobin Standardization Program. The HbA1c values obtained by the point-of-care analyzer and immunoturbidimetric assay used in our laboratory (Tina Quant II assay, Roche Diagnostics, Germany) yielded a significant correlation (r=0.887, p<0.0001).

Concurrently, capillary random plasma glucose was also measured using a glucometer (Accu-Check Performa, Roche Diagnostics, Mannheim Germany), calibrated to infer venous plasma glucose values [12]. Glucose estimations done with the glucometer and in the central laboratory using hexokinase method yielded a correlation coefficient of 0.89, p<0.0001. The testing devices were calibrated each day at the worksite and quality control was performed from time to time. Prescreening and screening tests were done on the same day 30 to 45 minutes post breakfast. RBG values were available for 2835 individuals.

### Statistical analysis

Correlation of RBG with HbA1c was computed using multiple linear regression equation corrected for age and gender. The optimal cut-off values for RBG corresponding to HbA1c values of 5.7% (39 mmol/mol), (cut-off for prediabetes) and ≥ 6.5% (48 mmol/mol), (cut-off for diagnosing diabetes) were computed using the receiver operating characteristic curve (ROC). The sensitivity and specificity of RBG for detecting diabetes and prediabetes based on HbA1c were calculated by the area under the curve (AUC). Diagnostic accuracy was calculated using the Youden’s index.

## Results

Prescreening was conducted for a total of 6030 persons, to verify eligibility for the prevention trial. Among them 2835, (men: 2226 women: 609; mean age 45.9 ± 5.6 years) with ≥ 3 risk factors for diabetes were eligible for the HbA1c and RBG testing. Table 1 shows the characteristics of the study cohort and the presence of risk variables. Of the risk factors, obesity (81.7%), sedentary lifestyle (63.2%) and abdominal obesity in men (60.6%) were predominantly noted.

**Figure 1:**
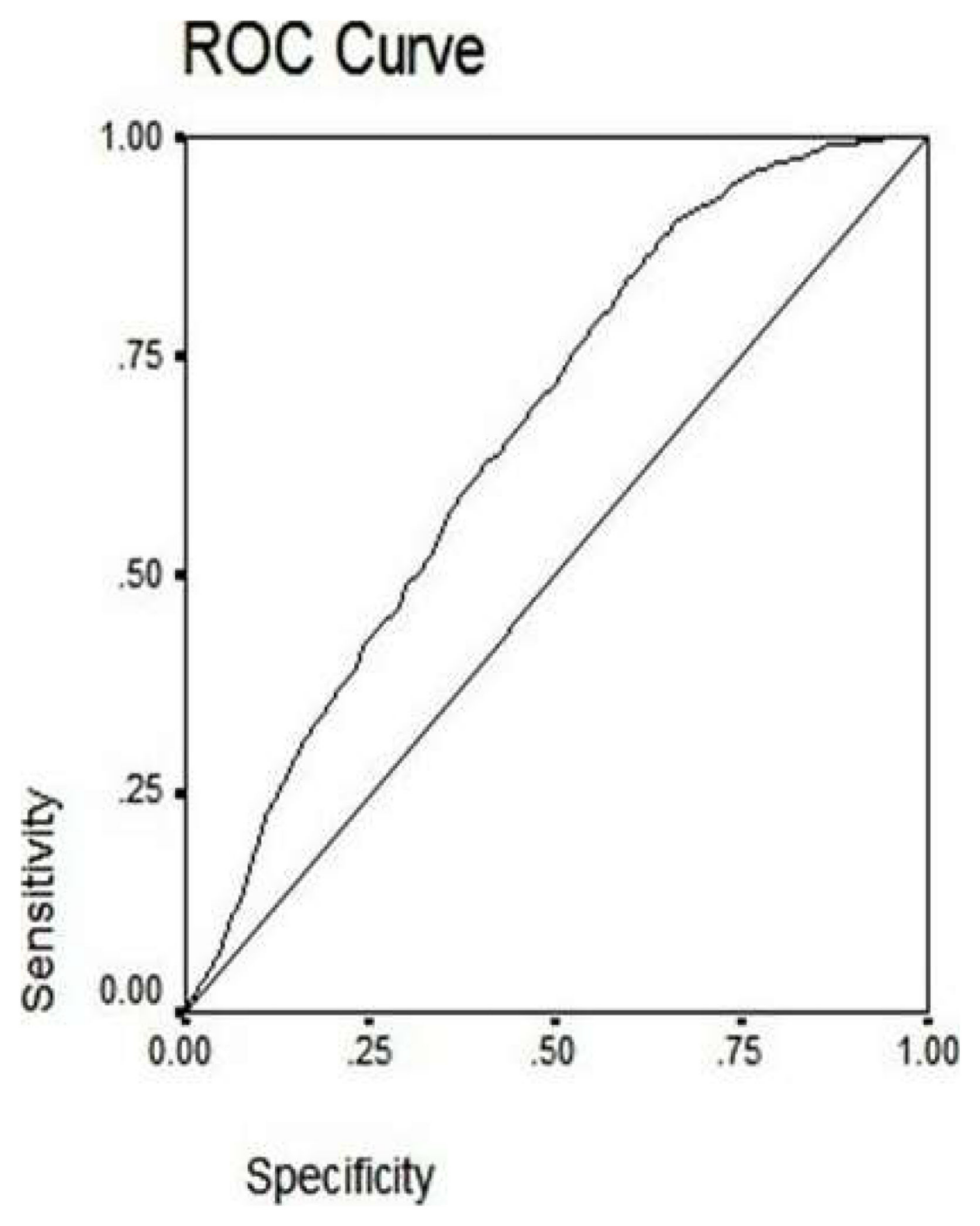
ROC showing predictive performance of RBG corresponding to an HbA1c value of 5.7% (39mmol/mol).

RBG showed significant correlation with HbA1c (r=0.40, p<0.0001). The regression equation was: y(HbA1c)=0.013*RBG+4.012. Using the ROC analysis, the cut-off value for RBG corresponding to an HbA1c of 5.7% (39 mmol/mol) (n=1121) was 113.5 mg/dl (6.3 mmol/L) (sensitivity 61%, specificity 61%, p<0.0001).The AUC was 0.662 ± SE 0.10 (95% CI 0.642-0.682), (Figure 1).

Cut-off value for RBG was 113.5 mg/dl (6.3 mmol/L) (sensitivity 61%, specificity 61%, p<0.0001). AUC = 0.662 ± SE 0.10 (95% CI 0.642-0.682). ROC: Receiver Operating Characteristic Curve; RBG: Random Blood Glucose; AUC: Area Under the Curve.

**Figure 2:**
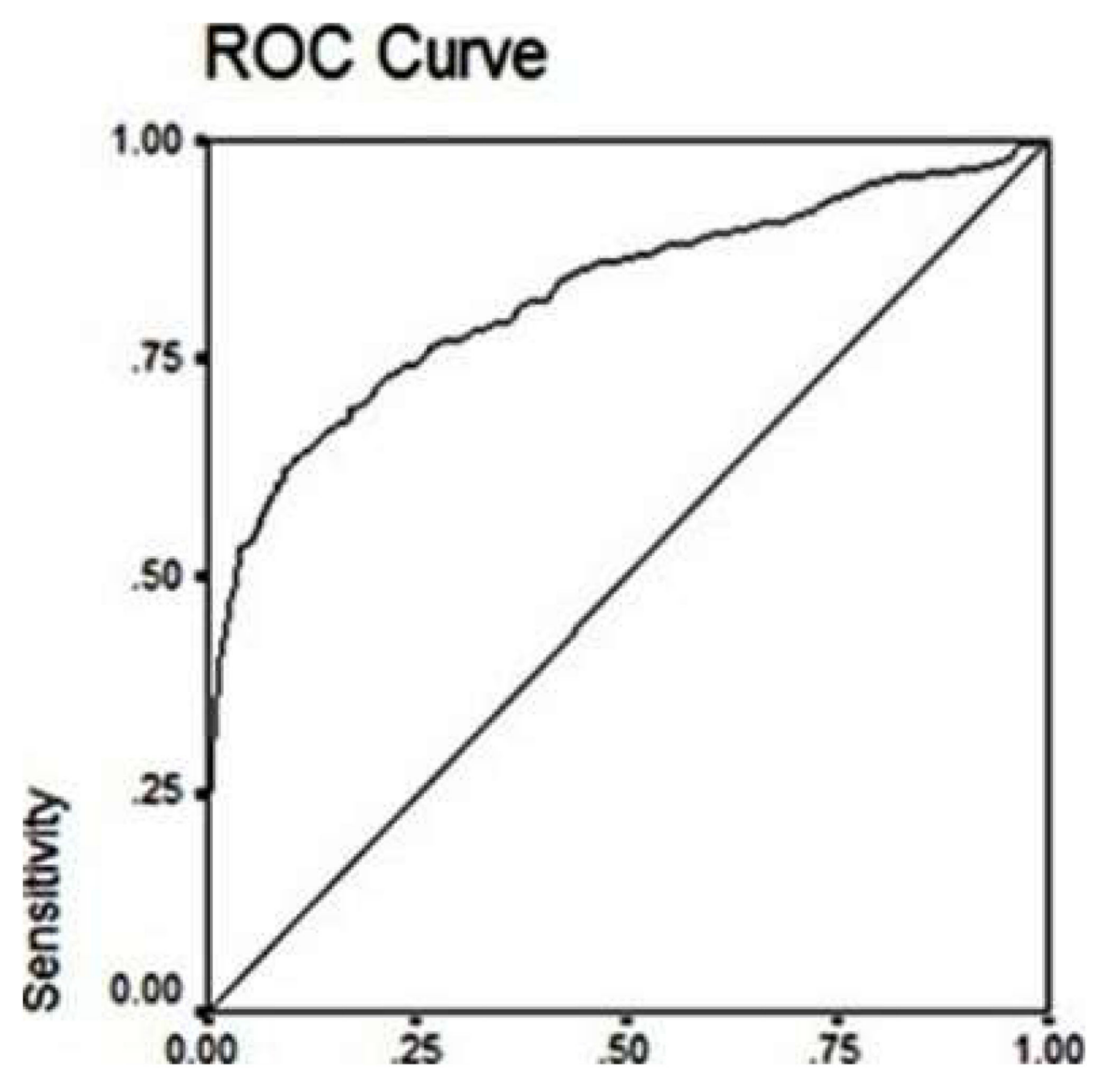
ROC showing predictive performance of RBG corresponding to an HbA1c value of 6.5% (48 mmol/mol).

Figure 2 shows that the cut-off value for RBG corresponding to HbA1c of 6.5% (48 mmol/mol) (n=283) was 140.5 mg/dl (7.8 mmol/L) (sensitivity 69%, specificity 83%, p<0.0000). The AUC was 0.823 ± SE 0.16 (95% CI 0.792-0.854). Based on the derived sensitivity and specificity, the Youden’s index wasabove 50%.

Cut-off value for RBG was 140.5 mg/dl (7.8 mmol/L) (sensitivity 69%, specificity 83%, p<0.0000). AUC = 0.823 ± SE 0.16 (95% CI 0.792-0.854). ROC: Receiver Operating Characteristic Curve; RBG: Random Blood Glucose; AUC: Area Under the Curve.

## Discussion

In this analysis, we intended to study the utility of RBG as a preliminary screening tool to identify persons at risk of developing diabetes and requiring further confirmatory diagnostic testing. The use of HbA1c test definitely has several advantages over that of blood glucose; but the higher cost of the test may limit its utility in large epidemiological studies. It may also be impractical in places where reliable test methods of HbA1c are not available. Therefore, the feasibility of using RBG in place of HbA1c or even a timed glucose measurement such as FPG or 2h PG values may be more advantageous and practical. In order to overcome this limitation, we assessed the cut-off values for RBG corresponding to the HbA1c values of 5.7% (39 mmol/mol) and 6.5% (48 mmol/mol) using ROC procedures. RBG values of 113.5 mg/dl (6.3 mmol/L) and 140.5 mg/dl (7.8 mmol/L) respectively were derived for prediabetes and diabetes defined by the above HbA1c values. However, it was noted that a RBG cut-off value of 113.5 mg/dl (6.3 mmol/L) did not show an acceptable level of sensitivity and specificity, and hence cannot be used to define prediabetes.

An earlier study conducted in the US, tested the performance of four screening methods for diabetes and dysglycemia based on risk score, capillary blood glucose measurement and a combination of both [6]. The study reported that a casual capillary blood glucose value of ≥ 140 mg/dl had a sensitivity of 65% and specificity of 96% for diagnosis of diabetes based on fasting and 2-hour glucose criterion. Similarly, a casual blood glucose value of ≥ 120mg/dl had a sensitivity of 62% and specificity of 90% for fasting plasma glucose of ≥ 110mg/dl.

Based on a comparison of RBG and corresponding oral glucose values, in a large number of randomly chosen individuals without a history of diabetes, it was suggested for Asian Indians with a RBG of >110 mg/dl (6.1 mmol/L) at screening can be recommended for definitive testing [8]. This research team, also from the same city had reported that RBG cut-point of 140 mg/dl (7.8 mmol/L) corresponded to the 2h PG ≥ 200mg/dl (11.1 mmol/L) used in diagnosis of diabetes. Values of 119 mg/dl (6.6 mmol/L) corresponded to the diagnostic value of 2h PG of ≥ 140 mg/dl (7.8 mmol/L) and 113 mg/dl (6.3 mmol/L) corresponded to impaired fasting glucose (ADA) value of ≥ 100mg/dl (5.6 mmol/L).

Our cut-off value for diabetes 140.5 mg/dl (7.8 mmol/L) does agree with the reported observations from the various studies discussed here. However, the derived cut-off value of 113.5 mg/dl (6.3 mmol/L) did not have acceptable sensitivity and specificity to identify prediabetes.

## Conclusion

The use of RBG testing with convenience of sampling at any time during the day could substantially reduce time and cost of screening both at a community level and in a clinical setting. However, the limitation lies in the accuracy of the glucose monitors used and other factors such as postprandial time and age that may influence the performance of the RBG test as reported by Rolka et al. [6]. It is also noted that the relation between blood glucose and the HbA1c value be influenced by several factors such as race and level of hemoglobin.

We report than an RBG value of ≥ 140 mg/dl (7.8 mmol/L) could be used to identify people who should be subjected to confirmatory OGTT to diagnose diabetes. RBG measurement may be applied if HbA1c or timed glucose testing are not practical for large scale screening of undiagnosed diabetes.

## Figures and Tables

**Table 1:** Baseline characteristics of study cohort.

	Total n=2835 Men: 2226 (78.5%) Women: 609 (21.5%)
**Characteristics (mean ± SD)**	
Age in years	45.9 ± 5.6
Weight (kg)	75.4 ± 11
Body mass index (BMI) (kg/m^2^)	27.7 ± 3.4
Waist Circumference (cm)	95.2 ± 7.9
Blood Pressure (mmHg)	
Systolic	129.3 ± 17.2
Diastolic	82.4 ± 10.7
HbA1c (%, mmol/mol)	5.9 ± 0.89, 41
Random Blood Glucose (mg/dl, mmol/L)	126.9 ± 42.8, 7.04
	
**Presence of Risk Factors n (%)**	
Positive Family History	1508 (53.2)
Overweight BMI 23.0-24.5 kg/m^2^	419 (14.8)
Obese BMI ≥ 25.0 kg/m^2^	2317 (81.7)
Abdominal obesity in men (≥ 90cm)	1718 (60.6)
Abdominal obesity in women (≥ 80cm)	577 (20.4)
History of Prediabetes	196 (6.9)
Hypertension – newly diagnosed (≥ 140/90mmHg)	663 (23.4)
Hypertension – Known	621 (21.9)
Sedentary Lifestyle	1793 (63.2)
Data presented are mean ± SD, n (%)	
